# A Multimodal SIMS/MALDI Mass Spectrometry Imaging Source with Secondary Electron Imaging Capabilities for Use with timsTOF Instruments

**DOI:** 10.1021/jasms.2c00381

**Published:** 2023-03-09

**Authors:** Kasper Krijnen, Joel D. Keelor, Sebastian Böhm, Shane R. Ellis, Claus Köster, Jens Höhndorf, Ron M. A. Heeren, Ian G. M. Anthony

**Affiliations:** † Maastricht MultiModal Molecular Imaging (M4i) Institute, Division of Imaging Mass Spectrometry, Maastricht University, 6229 ER Maastricht, The Netherlands; ‡ Bruker Daltonics GmbH & Co KG, Fahrenheitstraße 4, 28359 Bremen, Germany; § Molecular Horizons and School of Chemistry and Molecular Bioscience, University of Wollongong, Wollongong, New South Wales 2522, Australia

## Abstract

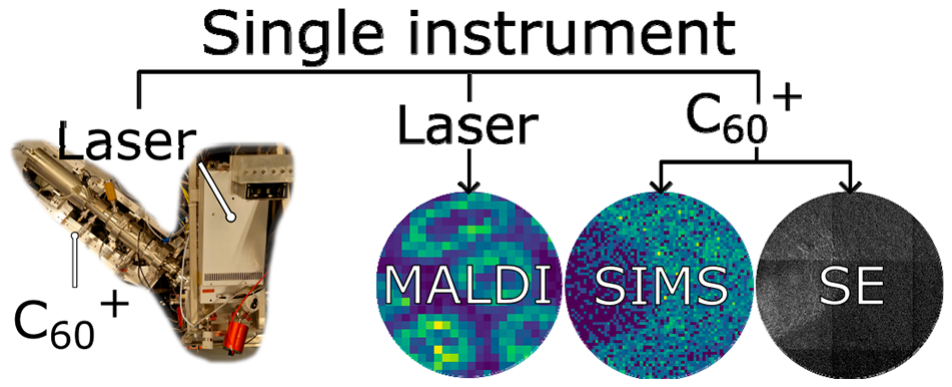

Mass spectrometry imaging (MSI) is a surface analysis technique that produces chemical images and is commonly used for biological and biomedical research. Multimodal imaging combines multiple imaging modes in order to get a more comprehensive view of a sample. Multimodal MSI images are often acquired using multiple MSI instruments, which leads to issues regarding image registration and increases the chance of sample damage or degradation during sample transfer. These problems can be solved by using a single instrument that can image in multiple modes. In order to improve the efficiency of multimodal imaging and investigate complementary modes of MSI, we have modified a prototype Bruker timsTOF fleX by adding secondary ion mass spectrometry (SIMS) and secondary electron (SE) imaging capabilities while preserving the ability to perform matrix-assisted laser desorption/ionization (MALDI). We show multimodal images collected on this instrument that required only trivial registration and were acquired without sample transfer between imaging trials. Furthermore, we characterize the performance of SIMS, SE, and MALDI imaging and compare the performance of the modified instrument to a commercial timsTOF fleX.

## Introduction

Mass spectrometry imaging (MSI) produces chemical images of a surface and is commonly used for biological and biomedical research.^1−5^ Two of the most popular MSI techniques are matrix-assisted laser desorption and ionization (MALDI) and secondary ion mass spectrometry (SIMS). With MALDI-MSI, a photoactive matrix is applied onto the sample, after which a laser beam probes the surface. Each laser shot produces ions that are detected using a mass analyzer and converted into a mass spectrum. These spatially resolved mass spectra can then be used to generate ion images of the surface. SIMS imaging is similar to MALDI-MSI, but uses an ion beam as a surface probe and generally does not require the addition of a matrix.^1^ MALDI is useful for the analysis of intact biomolecules, but usually has a larger pixel size (usually between 5–50 μm).^6,7^ Whereas SIMS produces, compared to MALDI, more elemental ions and fragments of larger biomolecules and usually has a smaller pixel size (usually between 50–2000 nm).^8−10^

Because MALDI and SIMS each excel in different areas, they can be used together to acquire complementary information.^11,12^ This complementary information is most often acquired in two separate, unimodal MALDI-MSI and SIMS imaging instruments.^13,14^ However, using separate instruments has drawbacks. Because images are made using separate instruments, extra coregistration steps are required, requiring more precision if the spatial resolution increases.^15,16^ Transfer between instruments can cost time and damage or degrade the sample. Because SIMS and MALDI instruments are often operated in different ways, separate personnel, training and software may also be required for the MALDI-MSI and SIMS imaging analyses. These drawbacks of unimodal instruments can be circumvented by multimodal imaging instruments.^17−19^

Multimodal instruments combine different complementary imaging modes into a single instrument. Multimodal instruments are often more expensive than unimodal instruments, but often less expensive than the two or more unimodal instruments they replace. Although multimodal instruments are gaining popularity, many are still built in-house with nonstandardized features, custom software, no commercial support, and lack integration with other instruments.^17^ Currently no commercially available multimodal mass spectrometer capable of producing both SIMS and MALDI mass spectral images exists.

In this manuscript we present a multimodal SIMS/MALDI instrument with secondary electron imaging capabilities. This instrument was made by integrating an Ionoptika 40 kV C_60_^+^ ion gun into a prototype Bruker timsTOF fleX mass spectrometer using only a few custom components. This instrument uses the original timsTOF fleX standardized sample transfer modules, control, and data analysis software and is compatible with existing sample preparation. Although not commercially available, such a modification could be applied to other timsTOF fleX instruments without disruption of existing MALDI workflows and using only commercially available software. This modification provides the benefits of multimodal SIMS and SE images in a top-of-the-line MALDI imaging mass spectrometer. Here, we demonstrate the multimodal capabilities of this instrument by analyzing biological tissue, generating SIMS, MALDI, and secondary electron (SE) images. The technical merits and limitations of this instrument are also discussed.

## Methods

### Materials

2,5-Dihydroxybenzoic acid (DHB, > 99%) and HPLC-grade methanol, ethanol, hexane, and chloroform were obtained from Sigma-Aldrich (Sigma-Aldrich, Zwijndrecht, NL). Blue and green acrylic paints were purchased from a local arts supplier (Maastricht, NL). Indium-Tin-Oxide (ITO) coated glass slides from Delta Technologies (Loveland, U.S.A.) were cleaned with lab-grade hexane and ethanol. Transmission electron microscopy (TEM) grids with hexagonal meshes of size 300 were obtained from Agar Scientific, Ltd. (Stansted, Essex, U.K.). Peptide calibration mixture II, “Pepmix II” was purchased from Bruker Daltonik, GMBH (Bremen, DE). Remaining mouse brain tissue samples from a different study were used.^20^

### Equipment

A 40 kV C_60_^+^ ion beam equipped with a “mosquito-tip” were purchased from Ionoptika, Ltd. (Chandler’s Ford, U.K.). The mosquito-tip upgrade is a restrictive aperture of the ion gun that enables a higher pressure difference between the gun outlet and gun main chamber at the expense of field of view (FOV, ∼3 mm FOV to ∼500 μm). A corresponding secondary electron detection (SED) power supply, raster support unit (RSU, model RSU2000 controller) controller, and accompanying software (Gun Controller version 3.0.3.5.73, Ionoptika SED version 1.1.9.1, and Raster Unit Control version 1.0.1.92, respectively) were also purchased from Ionoptika, Ltd. All SED and RSU hardware and software were used as-is, with the exception of the secondary electron detector.

A microchannel plate (MCP) detector was purchased from Hamamatsu Photonics Germany GmbH (model F9895, Gelder, DE) and used as a secondary electron detector in the source chamber. This MCP was installed and used as a SE detection unit without any modification or electrical adjustment.

A prototype timsTOF fleX instrument with a modified source chamber (modifications explained below), equipped with a turbomolecular pump (model HiPace 300, Pfeiffer Vacuum GmbH, Aßlar, DE) was provided by Bruker Daltonik. This modified source chamber provided SIMS imaging, MALDI-MSI, SE-imaging, optical stage viewing (using a supplied camera), and sample load-and-unload capabilities. However, the modified source eliminated the ease of switching between MALDI and electrospray ionization (ESI) capabilities that are usually present in the timsTOF fleX instruments. An ESI source was also provided for calibration and debugging purposes. This ESI source could be installed in place of the sample stage (used for MALDI-MSI and SIMS imaging), but was not used for any of the experiments recorded in this paper. An adapter flange between the source chamber and the ion gun and a table for mounting and positioning the ion gun were designed, machined, and assembled at Bruker Daltonik.

**Figure 1:**
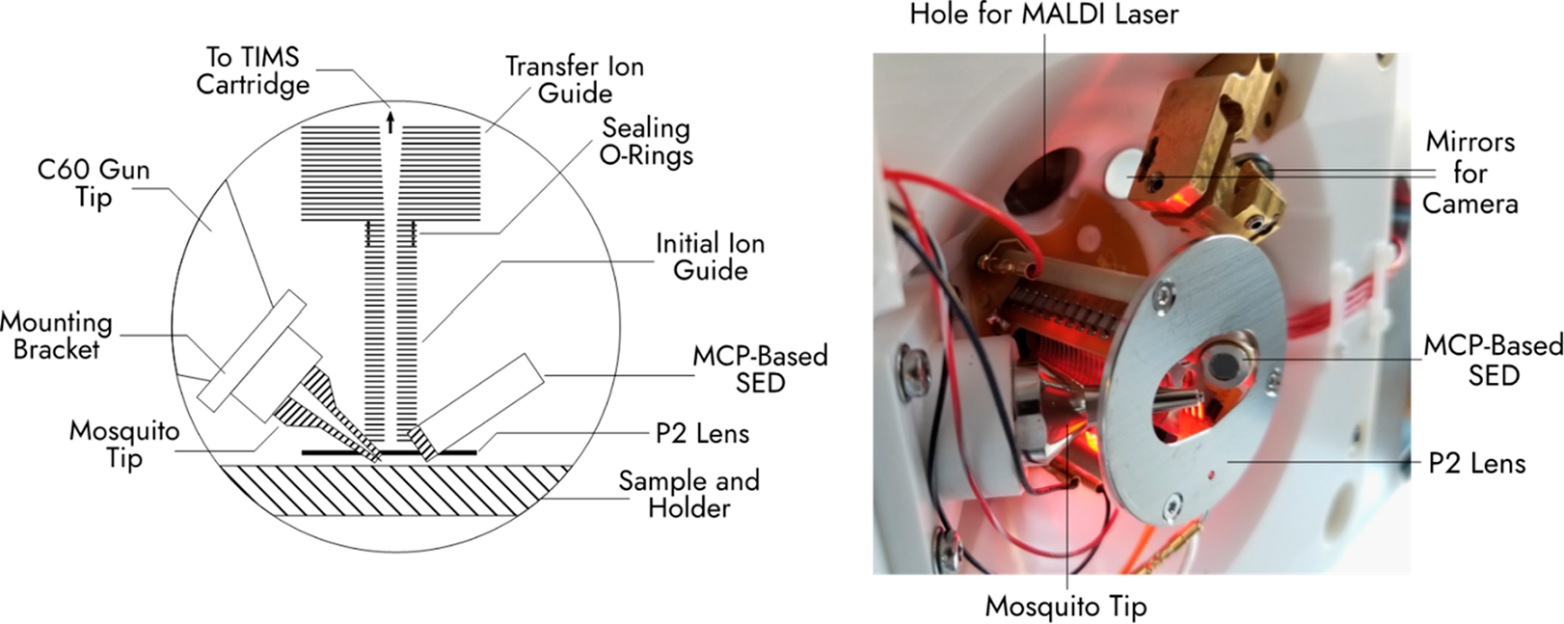
Scheme (left) and photograph (right) of the dual SIMS/MALDI source. The laser and C_60_^+^ ion beams enter the source through a hole (labeled at the top of the photograph) and the mosquito tip, respectively. The initial ion guide is composed of stacked electrodes with cutouts for the MCP-based SED, mosquito tip, LED, camera view, and laser beam to minimize their distances from the entrance of the initial ion guide and sample plate (not shown in the photograph).

For the modified source chamber, a custom, stacked-ring ion guide that allowed ion transmission from ions produced using both the stock timsTOF fleX laser and the added ion beam and also provided room for the MCP-based secondary electron detector (SED) was designed and manufactured in collaboration with Bruker Daltonik. See Figure 1 for a scheme and image of this ion guide and associated hardware. See Figure S1 of the Supporting Information for a scheme of the overall instrument and more details. Four 300 V DC power supplies (model ES 0300–045, Delta Elektronika B.V., Zierikzee, NL) were used to provide voltages to the sample plate, ion gun mosquito tip, and start and end of the stacked-ring ion guide, respectively. The four potentials within the ionization chamber (corresponding to the four DC power supplies) were set in a gradient of decreasing magnitude from the sample stage (highest magnitude) to the end of the custom ion guide (lowest magnitude) that leads into the trapped ion mobility spectrometry (TIMS) cell.

### Ion Gun Addition

#### SIMS Imaging

For all SIMS imaging experiments, the timsControl software (Bruker Daltonik, version 2.0) was used to provide method files and the flexImaging software (Bruker Daltonik, version 5.1) was used to provide stage motion for imaging. As primary ion beam control is not provided in the timsControl software, SIMS imaging was accomplished by first, ensuring that the ion beam is tuned and focused on the same location as the laser (see Figure S2). Second, turning “off” the laser in the timsControl software by turning the laser power and global attenuator to 0% (with this setting, the laser produces no laser ablation or ion signal). Third, setting the laser timing parameters to the desired SIMS irradiation time (for example, 1000 shots at 1 kHz laser repetition rate produces 1 s of SIMS irradiation time which corresponds to an approximate ion dose of ∼10^15^ ions per cm^2^ which far exceeds the static limit of ∼10^13^ ions per cm^2^). Fourth, starting the imaging experiment using the flexImaging software in a manner identical to that as when using the laser. Note that due to this method of imaging as well as the accumulation-based nature of the timsTOF fleX imaging mass spectrometer, all SIMS and secondary electron (SE) imaging experiments were in direct current (DC) mode and showed evidence of surface alteration, indicating a dynamic SIMS mode of action.^21^

#### SE Imaging

For SE imaging, ion gun settings were optimized for maximum current and focus on the conductive ITO glass slide with an aperture size of 100 μm. The ion beam was set to raster mode with a dwell time of 50 μs. Images were made using the Ionoptika Raster Unit Control software (version 1.0.1.92).

For “stitched” SE imaging of large areas, mouse and keyboard emulation was used to start the measurement, save the created image, and move the stage in predetermined 300 μm steps. 1122 images, each of an area of approximately 375 × 375 μm^2^ with a resolution of 1024 × 1024 pixels were stitched together with an offset of approximately 275 pixels. Stitching was performed using Python (Python, version 3.9, Python Software Foundation).

### Sample Preparation

#### Sectioning

A fresh-frozen mouse brain was sectioned using a cryostat microtome (Leica, Nussloch, Germany) in the sagittal plane (approximately 35% of the depth of the brain) at −20 °C with a thickness of 10 μm for imaging experiments and 10, 20, and 30 μm for ion beam induced sample surface ablation experiment, respectively. All sections were thaw-mounted on conductive, ITO-coated glass slides. After sectioning, the sample slides were stored at −80 °C. For measurement, the slides were (1) removed from the freezer; (2) immediately placed in a dry, sealed slide transport chamber with desiccant in the bottom to prevent condensation; (3) allowed to warm to room temperature; and (4) dried in a separate desiccating chamber for 10 min.

#### Laser Desorption Calibration

1.0 g of blue and 1.0 g of green acrylic paints were each diluted to 10 mL total volume with ethanol and allowed to settle. 0.2 mL of the blue and green supernatants were respectively pipetted and spread homogeneously over areas measuring ∼15 cm^2^ on a ground-metal MALDI sampling holder and allowed to dry. These sampling areas were used for source-voltage MALDI/LDI calibration studies (Figure S3) as they provided more consistency between laser shots than MALDI dried-droplet and matrix-sprayed areas.

#### SIMS Calibration and Preparation

The surface of a cleaned ITO slide was used as a sample for source-voltage SIMS and EM calibration and optimization. Rastering the primary ion beam in a 500 × 500 μm^2^ area of a cleaned ITO slide allowed for a consistent, stable signal for up to 30 min to be acquired, which facilitated easy mass calibration on peaks arising from the ITO slide, for example, *m*/*z* 656.59 for In_5_H_2_O_5_^+^ and 674.52 for In_5_H_4_O_6_^+^. Although the beam is rastered for SED-based imaging and for stable calibration signal, the mode of image production for SIMS is not through beam rastering but though stage motion.

For multimodal SIMS-secondary electron (SE) imaging and experiments, the sectioned samples were thawed, dried for 10 min in a desiccator, and then sputter-coated with a 1.0 nm layer of gold (SC7640 sputter coater, originally Polaron, Ltd., now Quorum Technologies, Laughton, U.K.).

### Imaging Parameters

#### Source Pressure

All MALDI, SIMS, and SE experiments in the main text were performed at a low source pressure (<5 × 10^–4^ mbar, hereafter “low pressure mode”). High source pressure (∼3.0 mbar, identical to the source pressure of an unmodified timsTOF fleX, hereafter “high pressure mode”) LDI is demonstrated and compared to low source pressure LDI (Figure S3). In comparison to most SIMS instruments, the source pressure in low source pressure mode is more than 3 orders of magnitude higher than most SIMS instrument sources. Most SIMS instrument sources operate in the molecular flow regime whereas the source in low pressure mode operates in the intermediate region between viscous and molecular flow. Both MALDI and LDI had greater sensitivity (between two and 20 times) in high pressure mode. High source pressure SIMS and SE experiments were attempted but no appreciable signal or images were able to be observed in either SIMS-mode or by the SE detector. Switching between high and low source pressures is possibly by switching on or off the turbomolecular pump connected to the ionization chamber, respectively. Approximately 10 min of equilibration is required to transition between high source pressures and low source pressures. Low source pressures with MALDI allow nearly instant transitioning between MALDI, SIMS, and SE experiments.

#### Multimodal MALDI-SIMS Imaging

For the multimodal MALDI-SIMS imaging experiments, for MALDI, a pixel size of 40 μm with a laser repetition frequency of 10 kHz with 2000 shots were used. For SIMS, a pixel size of 20 μm with an irradiation time of 1 s were used (at a C_60_^+^ current of ∼600 pA this corresponds to an ion dose of ∼9 × 10^14^ ions per cm^2^). In order to boost SIMS sensitivity, the C_60_^+^ ion beam was focused to an area of ∼5 μm^2^ and then scanned within each 20 × 20 μm^2^ pixel area. This scanning allows for a more “square” shaped pixel that is analogous to the 3D Smartbeam that similarly scans the laser beam to produce a square pixel.^22^ See Figure S2 for an example of the laser-based scan and alignment of the laser and C_60_^+^ beam. This “rastering” does not produce subpixel information. For pixels larger than 10 μm at exposure lengths of 1 or more seconds, this rastering of the primary ion beam produces slightly more consistent and higher intensity pixels than a focused, stationary primary ion beam as the total area of the pixel is able to be sampled.

For the alternating MALDI-SIMS experiments (Figure S4), fresh, ∼1.25 mm^2^ portions of the Bruker Daltonik-supplied rat tissue were imaged, respectively. In order to prevent sample alteration and degradation between images, all images of the rat tissue were acquired on the same day.

**Figure 2:**
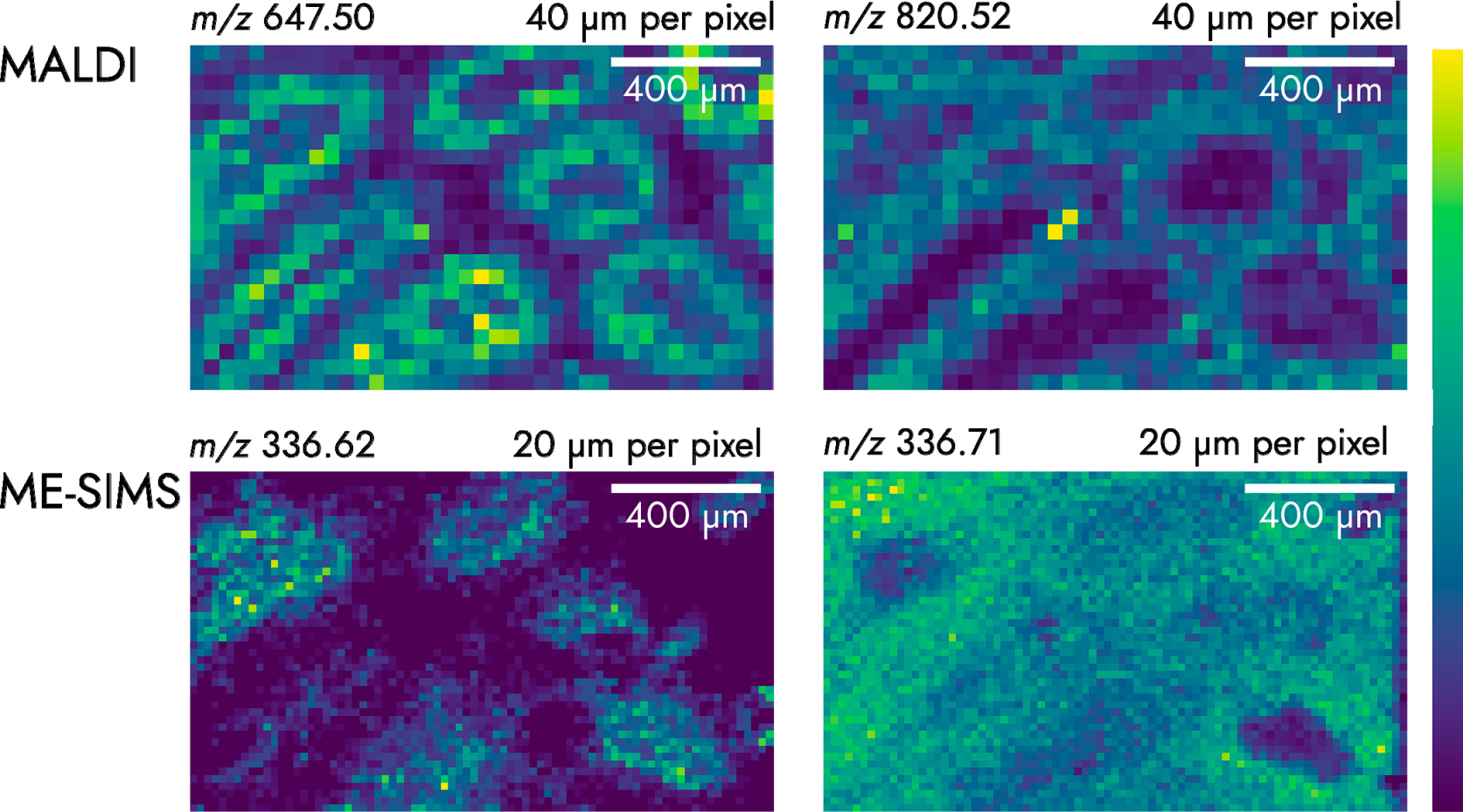
MALDI (40 μm pixel size) and ME-SIMS (20 μm pixel size) mass images acquired from the same tissue area of rat testis sections. Mass images are attributed to background correlated with the ITO slide (*m*/*z* 336.71), an inorganic species (*m*/*z* 336.62), and lipid species (*m*/*z* 647.50 and *m*/*z* 820.52). The two SIMS images are provided to demonstrate the advantages of a relatively high mass resolution of 30 000 at *m*/*z* 800 that can produce highly distinct visual distributions of inorganic ions. The MALDI ions were selected for images as they were relatively intense peaks that produced visually distinct spatial distributions. Additional discussion of peak identities and images are provided in Figure S7.

#### Multimodal SIMS-SE Imaging

For the multimodal SIMS-SE experiments (Figure S10), for SIMS, a pixel size of 10 μm with 1.25 s per pixel irradiation time (1250 shots and a frequency of 1000 Hz) was used. Primary ion gun voltages were optimized for maximum current using the 1 mm aperture (maximum observed was ∼900 pA with a usual current of ∼600 pA) onto the conductive ITO slide. For SIMS, the 300 μm primary ion beam aperture was used (which corresponded to a usual current of ∼200 pA), resulting in a spot size of approximately 5 μm (Figure S9). For SE, the 100 μm aperture was used, resulting in a pixel size of less than 1 μm.

**Figure 3:**
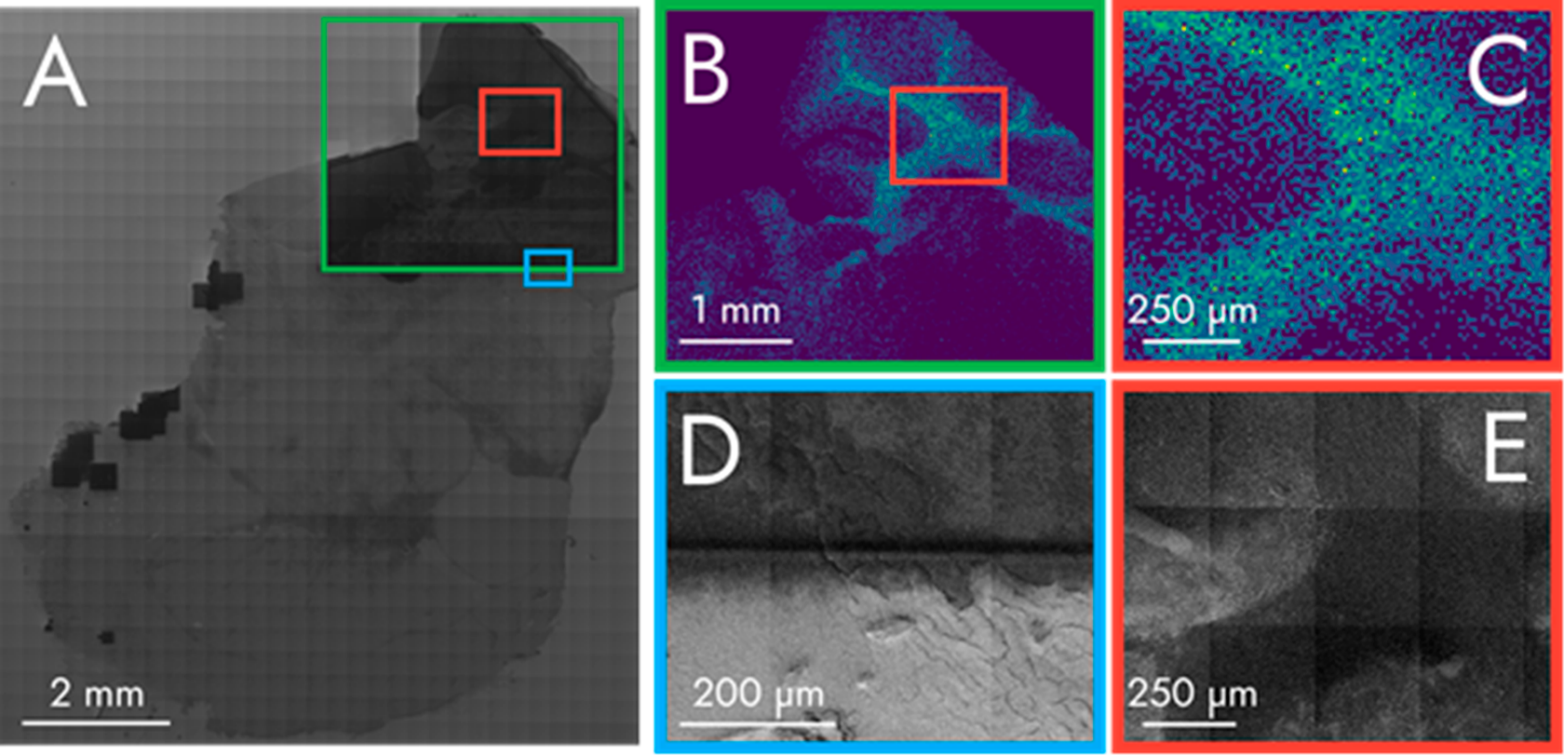
(A) stitched SE Image of a mouse brain tissue section with correspondingly colored and outlined squares indicating zoomed-in regions imaged with SIMS imaging of an unknown *m*/*z* at 628.57 (B, C) or SE imaging (D, E). Darker areas (visible in A, D, and E) were previously exposed to the C_60_^+^ ion beam prior to SE image acquisition. An SE image of the border of the SIMS imaging experiment can be seen in D. The same area within the SIMS imaging experiment is depicted in C and E.

All figures and data were acquired using the same instrument. The SE-images are secondary electron images that were produced using the primary ion beam and a secondary electron detector (SED), and not produced by a scanning electron microscope.

#### Ion beam Induced Sample Surface Ablation

Three random points were chosen on each of the 10, 20, and 30 μm-thick mouse brain sections and measured over time using the timsControl software in “ESI” mode (using the DC capabilities of the SIMS source, not using the ESI-source module). The “ESI” mode allowed acquiring chromatograms of total ion count (TIC) and an ITO-specific mass-to-charge (*m*/*z*) signal of 674.52 assigned to In_5_H_4_O_6_^+^). These chromatograms were acquired on each spot until no more signal was observed.

### Software

For MSI experiments, flexImaging and timsControl (Bruker Daltonik) were used. Python (version 3.9) was used in combination with the Bruker TDF-SDK API to create mass images from the produced.tsf files. Raster Unit Control (Ionoptika) was used to produce SE images. The timsControl software was used to move the stage between images. A Python script using the PyAutoGUI library was used to synchronize acquisition of consecutive SE images and then to stitch them together.

## Results and Discussion

### Multimodal MSI

We customized a prototype timsTOF fleX and equipped it with a C_60_^+^ ion gun and a SED, allowing the instrument to perform MALDI and SIMS measurements. All SIMS measurements are in “DC” mode, or dynamic SIMS, which can ablate the outermost surface layers. Matrix-enhanced SIMS (ME-SIMS) proved to be possible under these conditions, and protonated molecular signals are able to be observed between 200 to 1400 *m*/*z*. See Figure S6 for a comparison of ME-SIMS and MALDI mass spectra of a peptide mixture.

This modification to a MALDI instrument is beneficial because it allows the advancements in instrument workflow and ease-of-use commonly associated with MALDI imaging mass spectrometer to be used with SIMS, a technique that is often difficult to train users in and highly expensive for many instruments. Specifically, it allows for MALDI workflows to be adapted to SIMS without change in sample holder, instrument control, and data analysis software and without different sample preparation for ME-SIMS. Additionally, the use of a single sample stage and no need to transfer the sample between SIMS and MALDI instruments means that image coregistration for multimodal MALDI/SIMS images is not required as the coordinates system of the MALDI and SIMS-based pixels are identical if the beams are aligned. See Figure S2 for an example of such alignment. This identical coordinate system means that no errors or complicated algorithms involved in image registration are of concern for mul-timodal SIMS/MALDI imaging which can be simply overlaid (Figure S4).

To determine the optimal order of imaging modes, two multimodal MALDI-SIMS images were acquired. Figure 2 shows the results of such an imaging experiment on a rat testis section with MALDI first. After performing MALDI-MSI, the sample is still in a sufficient enough condition to obtain relevant, complementary SIMS images. The mass resolution is enough to distinguish the SIMS peaks with a 0.09 Da difference. The presence of ITO related peaks within the SIMS mass spectra likely indicates that the tissue section has been partially ablated and that the ITO surface is exposed.

The quality of the multimodal image was higher in all aspects with a MALDI-MSI first, SIMS imaging second approach. In the case of a MALDI-MSI second, SIMS imaging first, SIMS produced chemical noise (see Figure S8) that is attributed to matrix clusters and matrix cluster fragments. This chemical noise produced images with poor surface contrast and few spatially resolved features. The discrepancy between the enhanced ME-SIMS signal and the chemical-noise suppressed signal of SIMS imaging on a matrix-sprayed tissue may be due to the differences in MALDI matrices used. DHB was used as the matrix for the multimodal imaging to enhance lipid signal, whereas α-cyano-4-hydroxycinnamic acid (HCCA) was used for ME-SIMS and MALDI analysis of the peptide standard. Another possible reason for this discrepancy is that the chemical complexity of biological tissue has more interfering chemical species and chemical noise.

In the SIMS-first approach, the ablation of the MALDI matrix or other change to the surface by the ion beam appeared to slightly suppress the MALDI signal. In contrast, if MALDI was performed first it was identical to a unimodal MALDI-MSI experiment in signal and the ablation of the bulk of the MALDI matrix caused a reduction in the chemical noise and an increase in contrasting signal. However, after MALDI-MSI, ITO-related peaks may be observed, such as the peak at *m*/*z* 336.71, which we assign to a species that correlates with the background slide. We discuss the tentative identification of *m*/*z* values shown in Figure 2 in Figure S7.

In addition to enabling SIMS imaging capabilities, the addition of the C_60_^+^ gun enables rapid SE-imaging. The addition of SE imaging enables easy image and beam alignment of the primary ion beam and the laser together. Beam alignment is performed by using the mark left by the laser beam that is visible in SE mode. Using the SE image, the primary ion beam can be aimed specific pixels within the laser mark. Because the software used for MALDI and SIMS is the same and stage coordinates are retained in the imaging file after an imaging experiment, performing MALDI-MSI and SIMS imaging at different spatial resolution on exactly the same tissue area is possible, so long as the sample holder is not removed; which causes multiple-micrometer shifts in the locking position of the holder and of the ITO-coated glass slides. For a further explanation of this procedure, see Figure S2.

#### Multimodal SIMS and SE Imaging

Figure 3A contains a stitched SE image of a mouse brain section that had been sputter-coated with 1.0 nm of gold. Darker areas within the tissue have been irradiated by the C_60_^+^ ion beam prior to the SE image acquisition. This decrease in brightness after C_60_^+^ ion beam irradiation could be caused by carbon or ablated surface debris occluding the gold nanolayer, depletion of the gold nanolayer, or subsurface damage exposing the underlying tissue. The area within the green square has been previously imaged with SIMS imaging, an ion image of this experiment can be seen in Figure 3B.

Magnified SIMS imaging and SE images of the region in the red square (from Figure 3A) are shown in Figure 3C and 3E, respectively. The *m*/*z* 628.57 abundance correlates with the darker area in the middle of the SE image. We discuss the tentative identification of *m*/*z* 628.57 in Figure S7.

The edge of the SIMS imaging area is shown in Figure 3D. The brighter area corresponds to the region outside of the SIMS imaging experiment and had not been irradiated by the C_60_^+^ ion beam prior to the SE image. Features located outside the SIMS imaging experiment area can be observed continuing into the SIMS imaging experiment area, albeit with a decreased brightness, demonstrating that even after dynamic SIMS, the features of the surface remain intact enough to identify and correlate between MSI and SE imaging. The SE images also demonstrate the experimental spatial resolution the primary ion beam can obtain.

At the end of the SIMS imaging experiment, the C_60_^+^ ion beam was focused onto a single spot for over 30 min. An oval with shadowing effects can be seen at this position within the SE-image (Figure S10). The diameter of this oval is approximately 40 μm at its longest point, which is eight times larger than the diameter of the focused C_60_^+^ ion beam. If this is the impact crater of the primary ion beam, then this could mean that over time, the beam has a 40 μm variance reach. There is also an area darkened surrounding this oval, which goes outside the SIMS imaging area. One potential cause if this could be carbon or ablated surface debris occluding the gold nanolayer, even outside the sampling region.

#### Comparison with Unmodified timsTOF fleX

To compare the sensitivity and mass resolution of the modified timsTOF fleX prototype to an unmodified timsTOF fleX we took MALDI-MS measurements in quadruplicates of a homogeneous layer of acrylic paint with different instruments. The average spectra as well as an overlay to compare peak width can be seen in (Figure S5).

The unmodified timsTOF fleX showed higher abundances for ions below *m*/*z* 700 than the C_60_^+^ ion gun modified timsTOF fleX prototype. Whereas the most abundant peak of the unmodified timsTOF flex is *m*/*z* 275.39 with a signal-to-noise ratio (S/N) of 19 745, the most abundant peak of the modified timsTOF fleX prototype is *m*/*z* 984.45 with a S/N of 12 441. This abundance shift is likely due to differences in the respective tune files of the two instruments but may also indicate a bias for higher *m*/*z* values in the custom RF ion guide (Figure 1).

The mass resolution of the *m*/*z* 984.45 peak for the modified and unmodified timsTOF fleX instruments are 25 438 (*m*/Δ*m*_50%_) and 49 720 (*m*/Δ*m*_50%_) respectively. This could be due to the age of the modified timsTOF fleX prototype, differences in calibration and tuning or a combination thereof.

The addition of the SIMS capabilities does not affect the MALDI imaging capabilities, however not all MALDI-capable settings are possible with SIMS. In particular, the pressure required for SIMS is not compatible with the existing trapped ion mobility spectrometry (TIMS) as a higher pressure is required in the source than the exit of the TIMS ion optical element. Similarly, MALDI at low pressure is not possible with TIMS.

Unlike TIMS, tandem MS in the form of collision induced dissociation (CID) is possible with both SIMS and MALDI in low-pressure mode. However, the resulting performance is poor as a larger isolation window (for example, 10 *m*/*z*) must be used or the resulting signal is not sufficient for high quality fragment spectra. The authors attribute the need for a higher isolation window to the lack of collisional cooling in the source and intermediate ion optics before the collision cell due to the higher vacuum requirements of SIMS (for example, the use of low source pressure mode). See Figure S6 for tandem MS data of bradykinin 1–7 with ME-SIMS. Due to the need for specific optimization and high isolation window width, CID is best performed with SIMS in nonimaging modes or in imaging modes at very low spatial resolution (for example, > 50 μm per pixel).

### Ion Beam Induced Sample Surface Ablation

**Figure 4:**
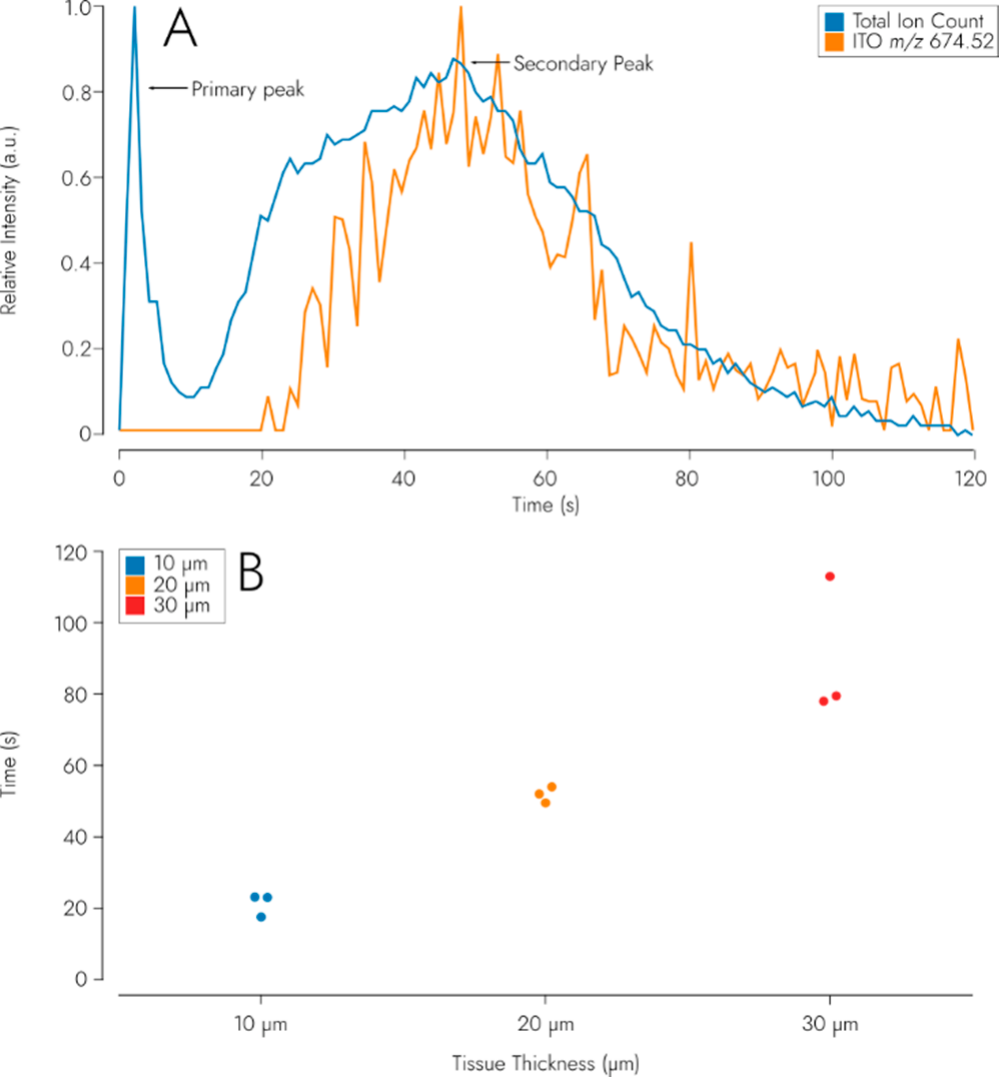
(A) Total-ion current (TIC) and Indium Tin Oxide peak (*m*/*z* 674.52) relative intensity over time on a 10 μm brain section. (B) Time in seconds it takes the ITO peak to surpass 1 × 10^3^ intensity for 10, 20, and 30 μm brain sections.

To determine the rate of sample surface ablation caused by the DC C_60_^+^, we monitored the total secondary ion current as well as the *m*/*z* 674.52 peak (attributed to In_5_H_4_O_6_^+^) that observed on clean ITO-slides over time while irradiating the surface of a mouse brain that was sectioned at thicknesses of 10, 20, and 30 μm with the C_60_^+^ ion beam (Figure 4). For all different slide thicknesses, a primary peak in the TIC is observed within the first three to five seconds of irradiation. During the primary peak there is no ITO signal.

The average time it takes for the ITO-related peak (*m*/*z* 674.52) to surpass 1 × 10^3^ intensity for 10, 20, and 30 μm brain sections is approximately 20, 50, and 90 s, respectively. The increase in ITO signal correlates with an increase in the TIC signal. The ITO signal indicates that at least a portion of the tissue sample has been ablated, exposing the ITO layer below.

## Conclusions

We have developed a multimodal instrument capable of MALDI-MSI, SIMS imaging, and SE imaging. This instrument allows for seamless data integration and easy imaging alignment due to the ability to align the laser and ion gun on the shared stage. Furthermore, this platform required few custom modification and is feasible to adapt for existing commercial mass spectrometers and allows for easy sharing of SIMS and MALDI imaging data. The limited number of modifications, the commercial control and analysis software, file formats, and sample transfer devices make this modified timsTOF fleX easier to use than multiple unimodal instruments. Using this platform, we performed multimodal SIMS/MALDI and SIMS/SE imaging without the need to remove the sample from the instrument. Additionally, we characterized the performance and sample surface ablation over time by the primary ion beam. In order to determine the effect of our modification, we compared our results obtained by a commercial, unmodified timsTOF fleX and found that, with proper tuning, comparable S/N can be achieved on both systems. However, the commercial timsTOF fleX usually provides a higher mass resolution (∼45 000 and ∼25 000 *M*/Δ*M*_50%_ at 400–1500 *m*/*z* for the commercial and multimodal systems, respectively) and greater ion sensitivity across a wider mass range. We expect that this instrument, due to the ease of coregistration, will be beneficial for correlative SIMS/MALDI/SE imaging. We anticipate this instrument and its future developments to enable high resolution imaging in all three modes without sample transfer, which will allow one to gain more complete information about a sample. Furthermore, we anticipate this instrument and others like it to provide a deeper understanding in the mechanisms of MALDI and ME-SIMS.

## Supporting Information Available

S1: Schemes and images of the multimodal SIMS/MALDI imaging mass spectrometer; S2: Depiction of the alignment of laser and primary ion beam; S3: Comparison of source voltage optimization at high and low pressures; S4: Overlay of MALDI/SIMS data; S5: Comparison of LDI signal on a commercial timsTOF fleX and the prototype timsTOF fleX with custom source; S6: ME-SIMS and MALDI spectral comparison of a calibration standard; S7: MALDI and SIMS tentative identifications; S8: Comparison of multimodal MALDI/SIMS imaging order; S9: Multimodal SIMS and SE image of a grid showing imaging pattern; and S10: SE image of ion beam induced sample damage (PDF)
