# Supplementary material for: A Multimodal SIMS/MALDI Mass Spectrometry Imaging Source with Secondary Electron Imaging Capabilities for Use with timsTOF Instruments
Source: J Am Soc Mass Spectrom. 2023 Mar 9;34(4):720–7. doi: 10.1021/jasms.2c00381 (PMC10080675; doi:10.1021/jasms.2c00381)
Supplement: Supplementary file 1 — js2c00381_si_001.pdf [file js2c00381_si_001.pdf]

Supporting Information

A Multimodal SIMS/MALDI Mass Spectrometry Imaging Source with Secondary Electron Imaging Capabilities for Use with timsTOF Instruments

Kasper Krijnen[1], Joel D. Keelor[1], Sebastian Böhm[2], Shane R. Ellis[1,3], Claus Köster[2], Jens Höhndorf[2], Ron M. A. Heeren[1*], Ian G. M. Anthony[1]

Affiliations:

[1] Maastricht MultiModal Molecular Imaging (M4i) Institute, Division of Imaging Mass Spectrometry, Maastricht University, 6229 ER Maastricht, the Netherlands.

[2] Bruker Daltonics GmbH & Co KG, Fahrenheitstraße 4, 28359 Bremen, Germany

[3] Molecular Horizons and School of Chemistry and Molecular Bioscience, University of Wollongong, Wollongong, NSW 2522, Australia.

Contents

S1: Multimodal SIMS/MALDI imaging mass spectrometer 2

S2: Alignment of the laser and primary ion beam 3

S3: Comparison of source voltage optimization at low and high source pressures 4

S4: Overlay of MALDI/SIMS data 5

S5: Comparison of LDI signal on commercial timsTOF fleX and the prototype timsTOF fleX with custom MALDI/SIMS source 6

S6: ME-SIMS and MALDI spectra of a calibration standard 7

S7: MALDI and SIMS tentative identifications 8

S8: Comparison of MALDI-first and ME-SIMS-first multimodal MALDI/SIMS imaging ... 9

S9: SIMS-produced mass image and corresponding SE image 10

S10: SE Image of C_{60}^{+} ion beam induced sample damage 11

References: 12

S1: Multimodal SIMS/MALDI imaging mass spectrometer

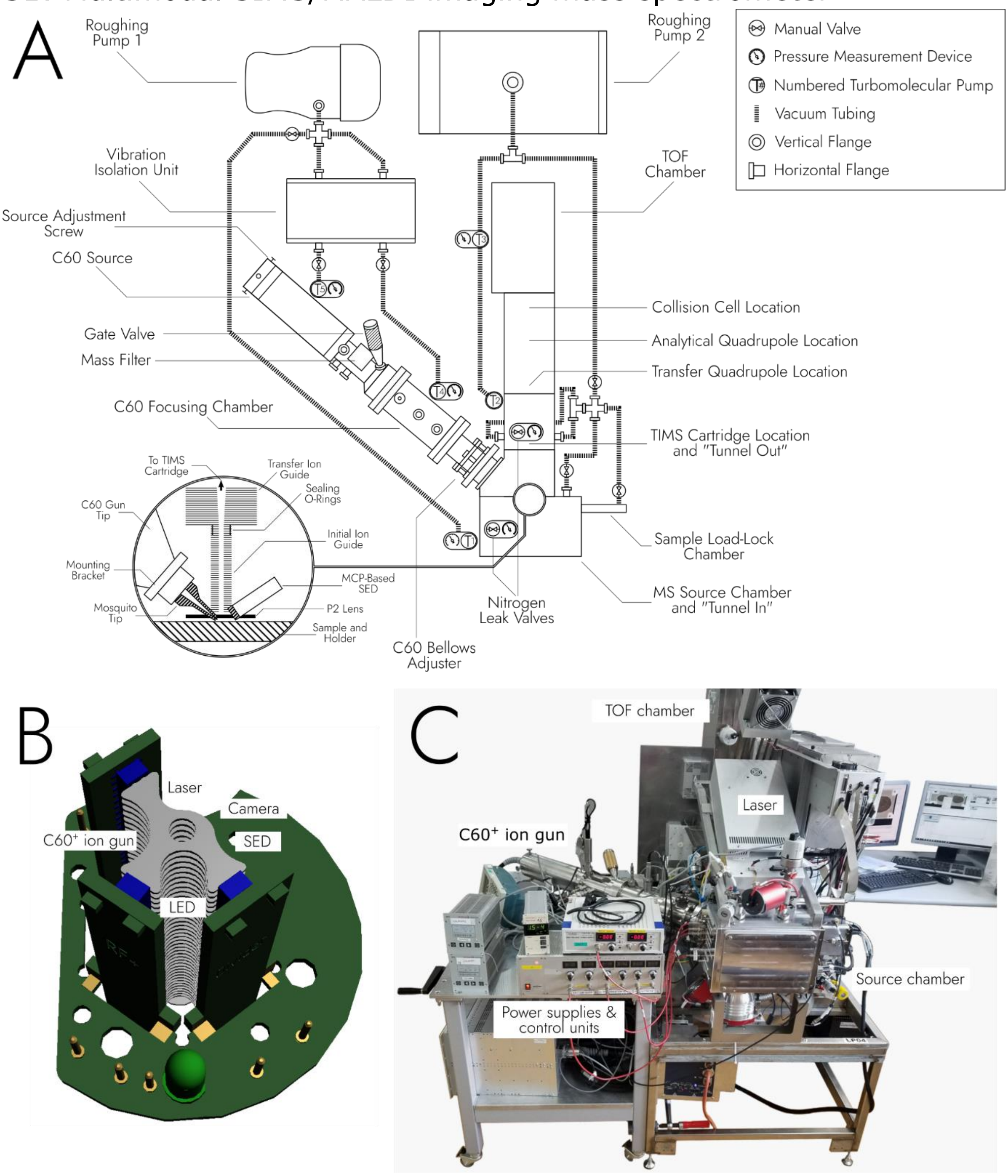

Figure S1: (**A**) A scheme of the vacuum chamber of the combined prototype timsTOF fleX with a $C60^{+}$ ion gun. A zoom-in of the imaging source ion optics and sample holder is provided in the bottom-left corner that is similar to the left panel in Figure 2. (**B**) A 3D-render of the initial stacked-ring ion guide and associated printed circuit boards (PCBs) of the ionization source optics. Labels indicate "cut-out" regions that allowed positioning of the ion gun mosquito tip, LED light, MCP-based SED, camera view, and laser beam. (**C**) A photograph of the (bare) instrument before transfer and final assembly. The vacuum chamber, ion gun, laser, and power supplies are labelled.

S2: Alignment of the laser and primary ion beam

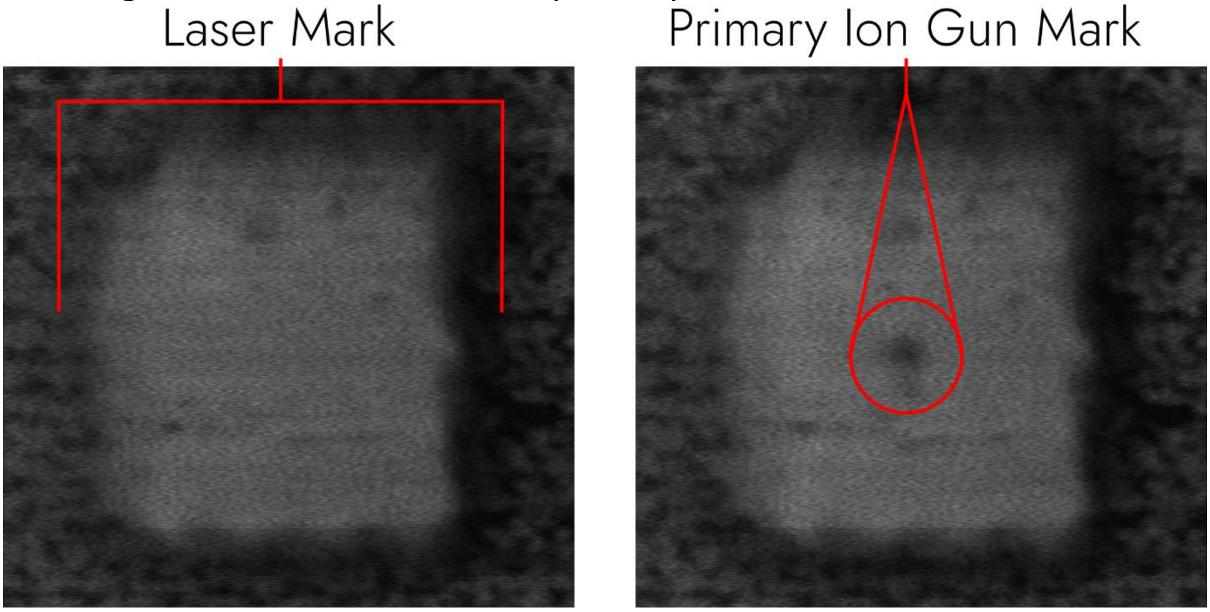

Figure S2: Alignment of the laser and primary ion beams is facilitated by the use of SE imaging. First, a laser mark (measuring approximately 50 micrometres on a side) and then imaged with SE imaging (left). Second, the primary ion beam (measuring approximately 5 micrometres diameter) is aimed at the centre of the laser mark and the surface is exposed for between two and ten seconds. Third, the area is re-imaged with SE imaging to verify the alignment accuracy (right).

S3: Comparison of source voltage optimization at low and high source pressures

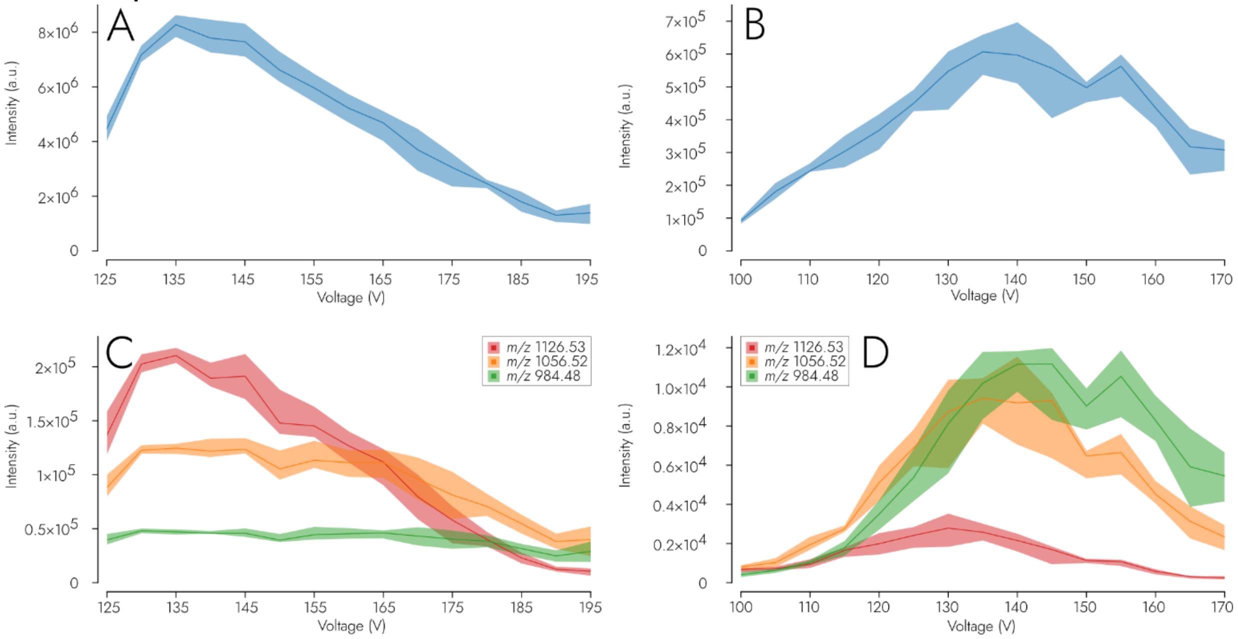

Figure S3: LDI-MS measurements of a homogenous acrylic paint layer at different source stage voltages at high and low source pressure ~3.0 mbar for the left column and ~3 × 10^{-4} mbar for the right column, respectively. TIC signal at different source stage voltages at high source pressure **(A)** and low source pressure **(B)**. In both high and low source pressure, the maximum TIC signal is at 135 V. The three most abundant peaks are shown in (**C**) and (**D**), respectively at high and low source pressures. Although the TIC maximum is the same between both source pressure modes, the relative intensities of the three most abundant ions is inverted. In (**C**), *m/z* 1126.53 is highest and in (**D**) *m/z* 984.48 is highest. This demonstrates a change in relative mass spectral intensity in the different pressure modes.

S4: Overlay of MALDI/SIMS data

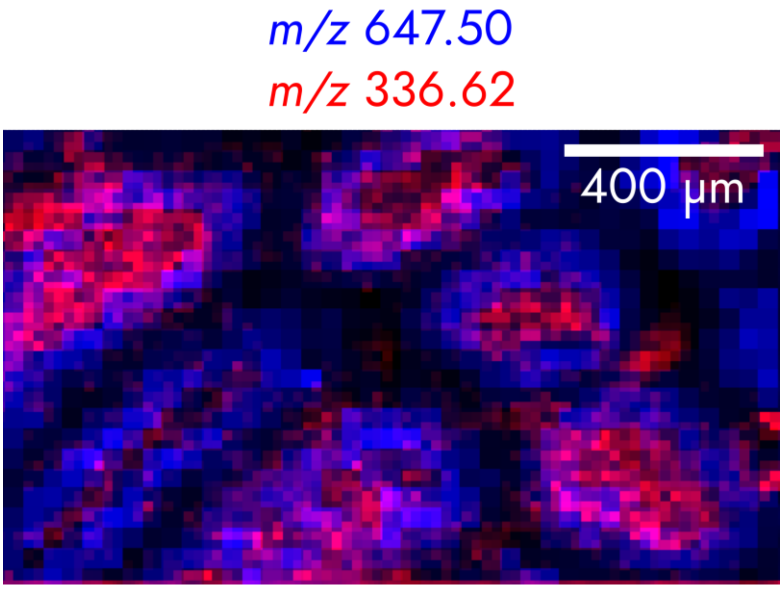

Figure S4: An overlay of the *m/z* 336.62 (in red, generated with SIMS) and *m/z* 647.50 (in blue, generated with MALDI) mass images that are also shown Figure 2 (left column). The overlay was performed using the coordinates of the modified, prototype timsTOF fleX stage. As the laser beam for the MALDI image was aligned with the ion beam for the SIMS image, the coordinates need no translation or adjustment as would be required in a two-unimodal instrument setup. Additionally, no sample exchange was performed between SIMS and MALDI image acquisition and thus sample transfer-based damage was avoided. Both data files were acquired using fleXimaging, of the Bruker .d file format (with .tsf and associated files within the .d file structure), and loaded into SCiLS for visualization. This overlay shows the inherent integration of the SIMS and MALDI data produced using the modified, prototype timsTOF fleX.

S5: Comparison of LDI signal on commercial timsTOF fleX and the prototype timsTOF fleX with custom MALDI/SIMS source

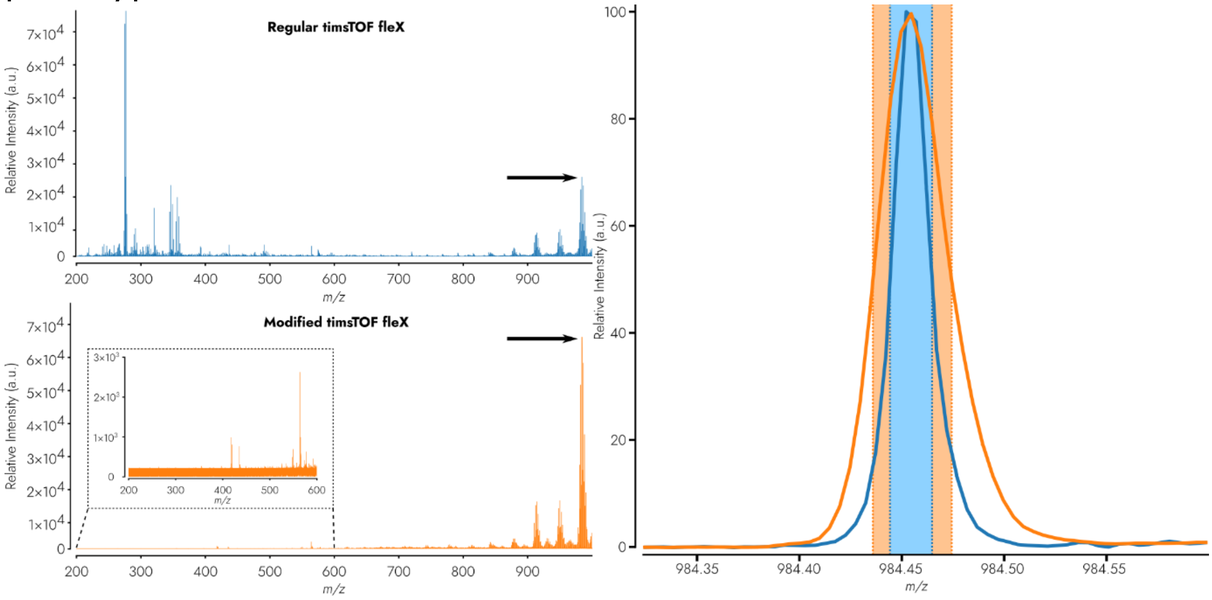

Figure S5: The left two sub-plots each show average mass spectra of four randomly selected LDI-MS measurements of a homogeneous layer of acrylic paint of both an unmodified timsTOF fleX (trace in blue, top) as well as the custom timsTOF fleX prototype (trace in orange, bottom). The spectrum from the unmodified timsTOF fleX shows higher abundances for ions below 700 *m/z* than the modified timsTOF fleX prototype and essentially no ion signal below *m/z* 400 that is above the noise level (see insert that shows a zoomed-in region between 200 to 600 *m/z*). This abundance shift is likely due to differences in the respective tune files of the two instruments but may indicate a bias for higher *m/z* values in the custom RF ion guide (shown in Figure 1). Arrows in the left two plots indicate peaks at 984.45 *m/z* that are overlaid and scaled to equal height in orange and blue traces for the modified and unmodified timsTOF fleX instruments, respectively. The FWHM is indicated by the areas with their corresponding colors. The resolution of the peaks in the right sub-plot from the modified and unmodified timsTOF fleX instruments are 25,438 ($m/\Delta m_{50\%}$) and 49,720 ($m/\Delta m_{50\%}$), respectively. After these results were collected, a detector optimization was performed on the unmodified timsTOF fleX instrument which achieved a mass resolution of ~30,000 $m/\Delta m_{50\%}$. The modified, prototype timsTOF fleX has not been able to achieve a mass resolution near the 49,720 $m/\Delta m_{50\%}$ of the unmodified timsTOF fleX. Although the source modification likely results in slightly altered performance of the timsTOF fleX, it is unlikely that this modification causes the reduction in mass resolution because the timsTOF fleX is an orthogonal TOF, meaning that the ion source is decoupled from the mass resolution. This can be seen in ESI-mode of a timsTOF fleX with comparison to MALDI mode.

S6: ME-SIMS and MALDI spectra of a calibration standard

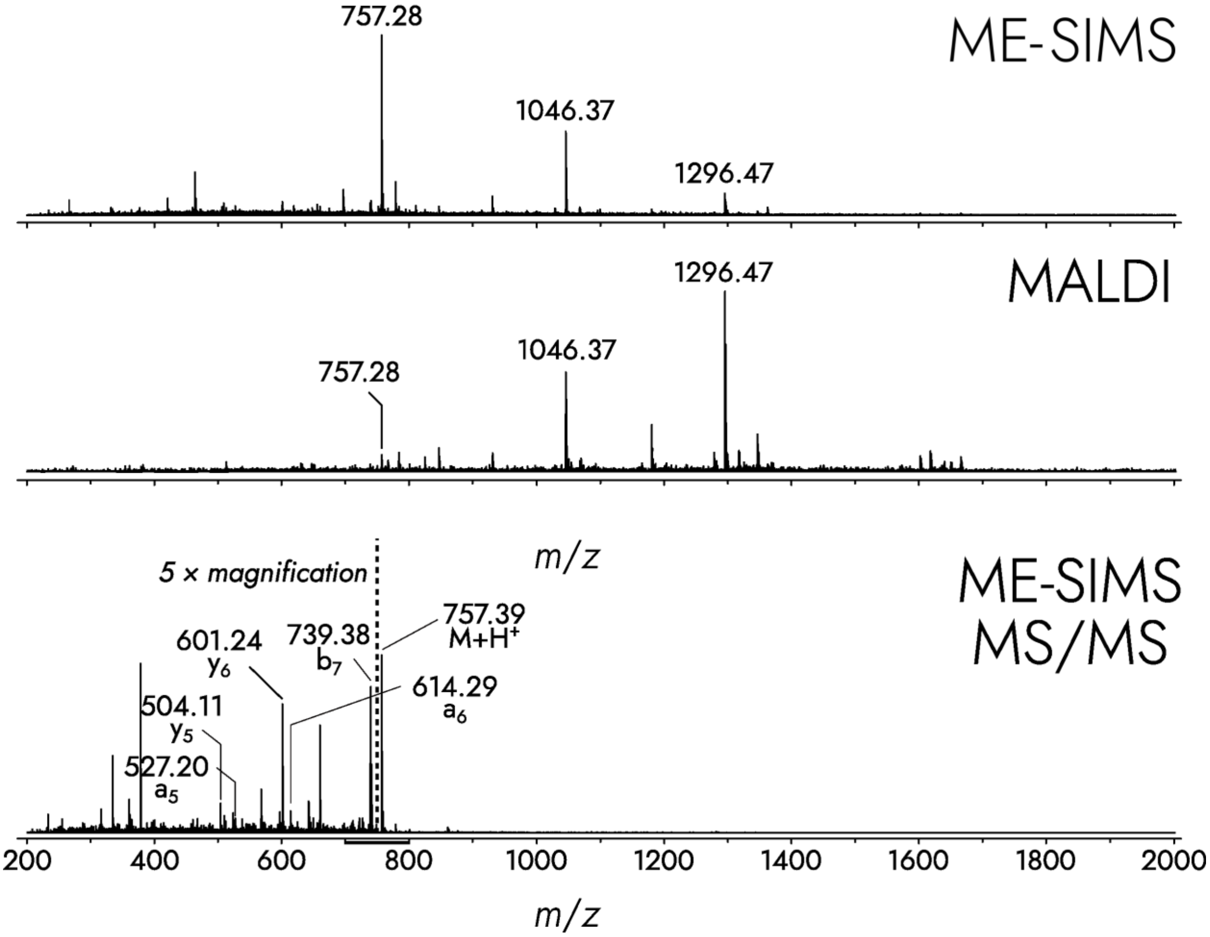

Figure S6: Mass spectra of Peptide Calibration Standard II "Pepmix-2" (Bruker Daltonik). Labelled *m/z* values of 757.28, 1046.37, and 1296.47 correspond to protonated peptides of Bradykinin Fragment 1-7, Angiotensin II, and Angiotensin I, respectively. The top two mass spectra were acquired on the prototype timsTOF fleX in positive ion mode within minutes of one another using the same source, the bottom mass spectrum was acquired at a later date using a different mass tune setting (using tune masses from the "Pepmix-2" calibration standard). Bradykin, for reference, has a theoretical m/z value of 757.399. As the mass spectra were acquired with the same mass analyzer, they had similar mass resolutions (approximately 25,000 m/Δm), mass ranges, and peak shapes. The top plot demonstrates that matrix-enhanced SIMS (ME-SIMS) is possible on the prototype timsTOF fleX and allows observation of *m/z* values of intact, protonated peptides at high relative intensity up to at least 1296 *m/z*, similar (although of a different relative abudance) to MALDI. The bottom plot is a tandem mass spectrum of ME-SIMS produced ions using the modified, prototype timsTOF fleX instrument. Some major fragments of bradykinin 1-7 (m/z 757.39) are identified in the bottom spectrum. The isolation *m/z* width and collision energy settings used for the bottom mass spectrum were 10 and 70, respectively. The bottom spectrum was normalized in intensity to the base peak (at 757.39 *m/z*). After this normalization, the peaks below 750 *m/z* were increased in height by five times to enable better visualization.

S7: MALDI and SIMS tentative identifications

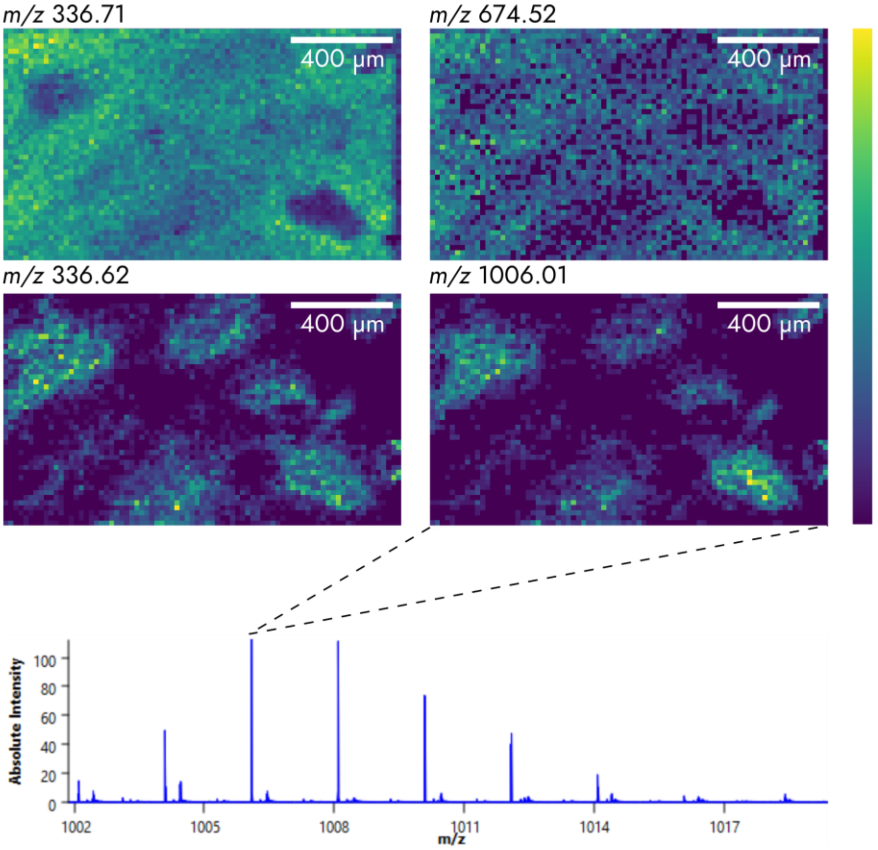

Assoc. Fig.	Tissue	Mode	*m/z*	Tentative ID	Theoretical m/z	Mass error (ppm)
Fig 2	Rat Testis	MALDI	647.503	SM d18:1/12:0 + H+	647.5122	-14.98041272
Fig 2	Rat Testis	MALDI	820.528	PC(36:4)+K+	820.5259	2.071841974
Fig 2	Rat Testis	ME-SIMS	336.71	ITO related*		
Fig 2	Rat Testis	ME-SIMS	336.62	Inorganic Cluster**		
Fig 3	Mouse Brain	Meta-SIMS	628.57	Cer 39:2;2 +Na+	628.563914	9.68238848

Figure S7: Reproductions of the *m/z* 336.71 and 336.62 images (top images, left column) from Figure 2 with visually correlated images (top images, right column). The *m/z* 336.71 is unknown and was not able to be confidently assigned but is spatially correlated to a peak within the same SIMS image, shown at *m/z* 674.52 which was identified as $In_5H_4O_6^+$ and is a common background peak of ITO coated slides. The presence of these peaks indicates that the C_{60}^+ beam was able to ablate and ionize the slide behind the tissue, which shows that much of the tissue had been ablated by MALDI-MSI or SIMS. The *m/z* 336.62 peak is presumed to be an inorganic cluster and corresponds to a cluster of inorganic peaks (that lack a carbon isotopic pattern) that have a maximum intensity at *m/z* 1006.01 (shown as a blue spectrum in the middle of Figure S7). Tentative identifications are made for each of the *m/z* values shown in Figures 2 and 3 at the bottom table of Figure S7. No identifications were made for 336.71 and 336.62, however these two peaks are similar in spatial distribution to more well characterized inorganic species, specifically ITO and a separate inorganic cluster, as indicated by the single and double asterisks, respectively. The reason these peaks were highlighted was due to their highly different spatial spatial distributions and their inorganic nature. The MALDI peak at 820.5276 is putatively identified as PC(36:4)+K^+ [1]. The MALDI peak at 647.503 is close in mass to SM D18:1/12:0+H^+ [2] and the SIMS peak at 628.57 close in mass to Cer 39:2;2+Na^+ [3]. However, these two peaks are not expected lipid species in this sample and thus no confident identification is made of these peaks.

S8: Comparison of MALDI-first and ME-SIMS-first multimodal MALDI/SIMS imaging

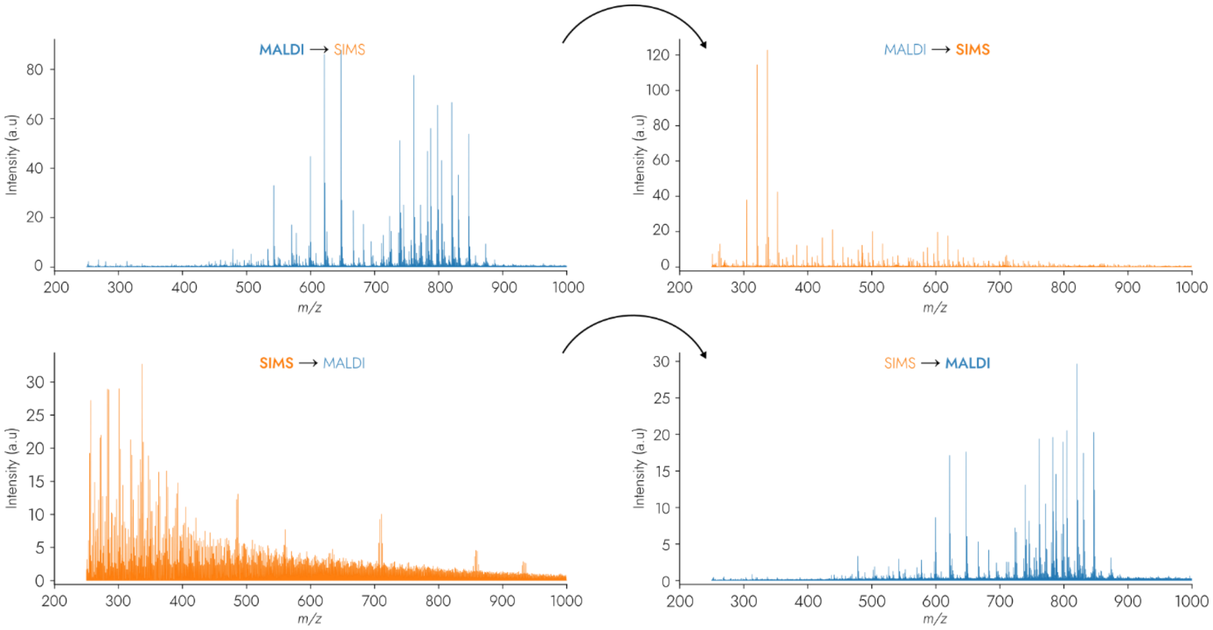

Figure S8: Four average mass specta of two multimodal image areas of the rat testes sample. The top mass specta correspond to imaging by MALDI then ME-SIMS; the bottom mass spectra correspond to imaging by ME-SIMS then MALDI. The spectra in blue are MALDI spectra the spectra in orange are ME-SIMS spectra. The sequence of MALDI then ME-SIMS produced superior results as the MALDI peaks are of higher intensity and the chemical noise (observable as a broad, low-intensity, repeating set of peaks in the lower-left plot) for SIMS are removed. However, some of the intense peaks in the SIMS spectrum post-MALDI (top right) are observed both outside and inside the tissue area, indicating areas of ablated tissue and sampling of the slide below).

S9: SIMS-produced mass image and corresponding SE image

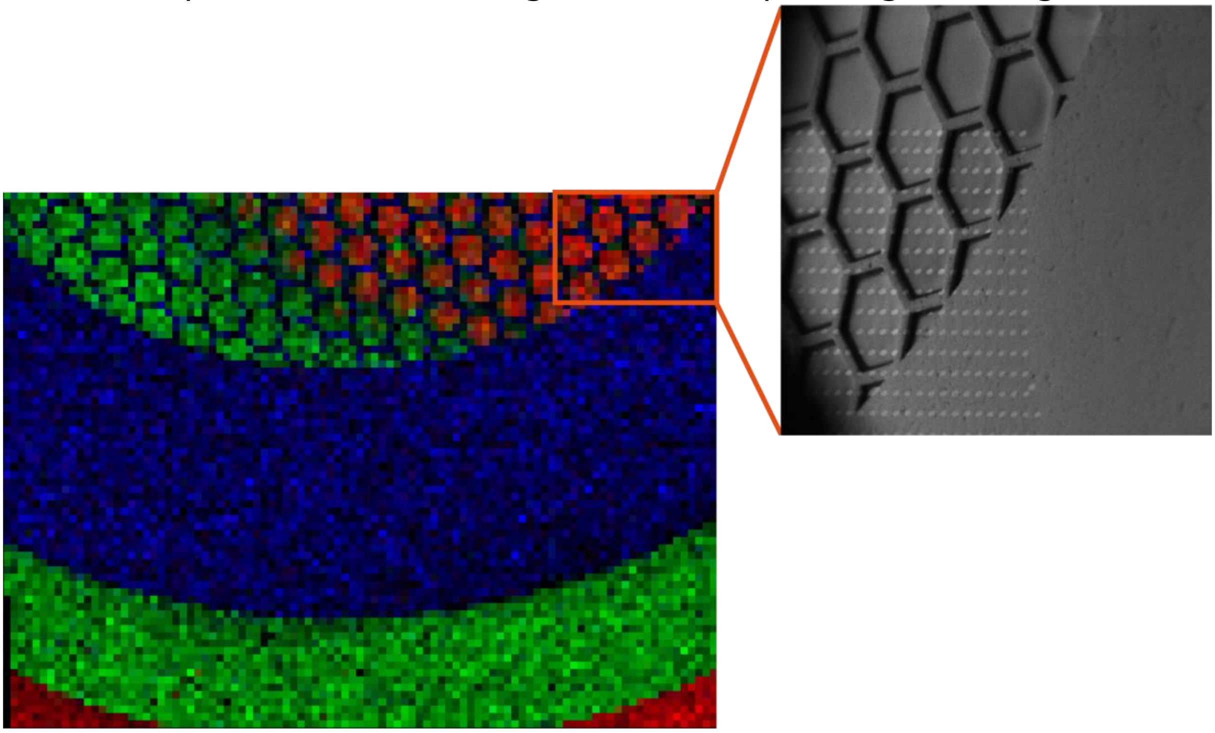

Figure S9: SIMS (left) and C60-produced SED (right) images of a TEM imaging grid over a dried spot of polyalanine on an indium tin oxide (ITO)-coated glass slide. The SIMS image red, green, and blue color values correspond to the normalized intensities of *m/z* values 367.7 (an ITO cluster), 569.3 (a fragment of polyalanine), and 609.8 (a mass associated with the TEM imaging grid), respectively. The spots shown in the SED image correspond to pixels in the top-right corner of the SIMS image and were measured at approximately 4 × 7 μm in size with a 10 μm step size between pixels at a rate of 3 pixels per second.

S10: SE Image of C_{60}^+ ion beam induced sample damage

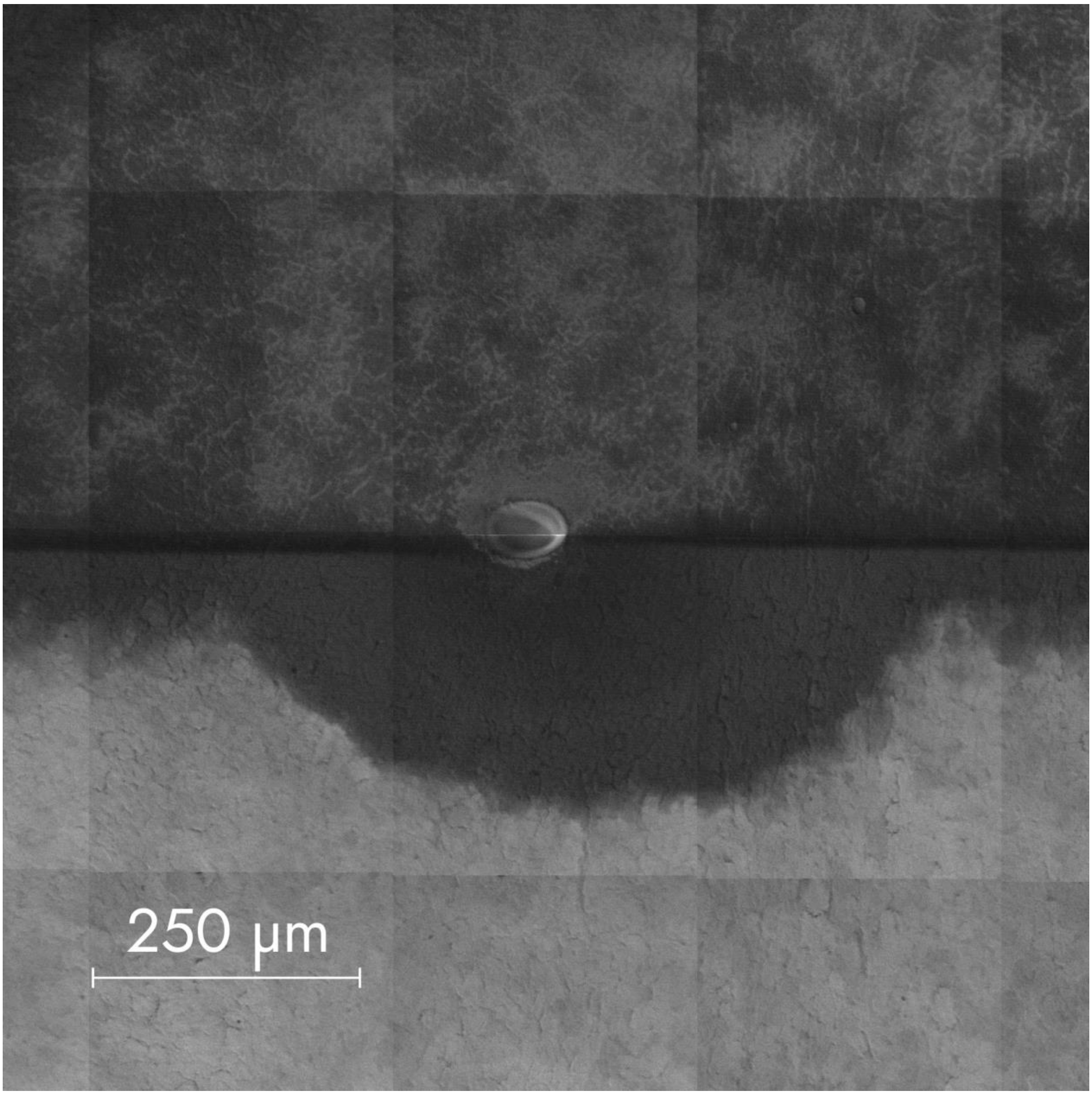

Figure S10: C_{60}^+ induced sample damage on rat brain section. The C_{60}^+ beam irradiated the section for approximately 30 minutes. Although the beam size was 5 µm, the hole has a diameter of 40 µm, indicating that the area of surface damage can exceed the beam diameter.

References:

1. Zemski Berry, K. A.; Hankin, J. A.; Barkley, R. M.; Spraggins, J. M.; Caprioli, R. M.; Murphy, R. C., MALDI Imaging of Lipid Biochemistry in Tissues by Mass Spectrometry. *Chemical Reviews* **2011,** *111* (10), 6491-6512.
2. Fahy, E.; Sud, M.; Cotter, D.; Subramaniam, S., LIPID MAPS online tools for lipid research. *Nucleic Acids Research* **2007,** *35* (suppl_2), W606-W612.
3. Pauling, J. K.; Hermansson, M.; Hartler, J.; Christiansen, K.; Gallego, S. F.; Peng, B.; Ahrends, R.; Ejsing, C. S., Proposal for a common nomenclature for fragment ions in mass spectra of lipids. *PLOS ONE* **2017,** *12* (11), e0188394.
